# ANDA: an open-source tool for automated image analysis of in vitro neuronal cells

**DOI:** 10.1186/s12868-023-00826-z

**Published:** 2023-10-24

**Authors:** Hallvard Austin Wæhler, Nils-Anders Labba, Ragnhild Elisabeth Paulsen, Geir Kjetil Sandve, Ragnhild Eskeland

**Affiliations:** 1 Institute of Basic Medical Sciences, Department of Molecular Medicine, Faculty of Medicine, University of Oslo, Blindern, 1112, 0317 Oslo, Norway; 2 Section for Pharmacology and Pharmaceutical Biosciences, Department of Pharmacy, University of Oslo, 0316 Oslo, Norway; 3 Department of Informatics, University of Oslo, 0316 Oslo, Norway; 4 PharmaTox Strategic Research Initiative, Faculty of Mathematics and Natural Sciences, University of Oslo, 0316 Oslo, Norway; 5 Centre for Cancer Cell Reprogramming, Institute of Clinical Medicine, Faculty of Medicine, University of Oslo, 0317 Oslo, Norway

**Keywords:** High-throughput imaging, Neuronal differentiation, Neurite morphology, Automated image processing

## Abstract

**Background:**

Imaging of in vitro neuronal differentiation and measurements of cell morphologies have led to novel insights into neuronal development. Live-cell imaging techniques and large datasets of images have increased the demand for automated pipelines for quantitative analysis of neuronal morphological metrics.

**Results:**

ANDA is an analysis workflow that quantifies various aspects of neuronal morphology from high-throughput live-cell imaging screens of in vitro neuronal cell types. This tool automates the analysis of neuronal cell numbers, neurite lengths and neurite attachment points. We used chicken, rat, mouse, and human in vitro models for neuronal differentiation and have demonstrated the accuracy, versatility, and efficiency of the tool.

**Conclusions:**

ANDA is an open-source tool that is easy to use and capable of automated processing from time-course measurements of neuronal cells. The strength of this pipeline is the capability to analyse high-throughput imaging screens.

**Supplementary Information:**

The online version contains supplementary material available at 10.1186/s12868-023-00826-z.

## Background

One of the defining characteristics of the central nervous system is the neuronal interconnectivity which facilitates the cell-to-cell communication required for normal brain function [1]. Establishment of neuronal networks in the developing brain are constituted by neuronal connections and can be influenced by the surrounding glia [2]. Neural development is a spatiotemporally fine-tuned biological process that spans the genesis of neurons to the maturation of functional neural tissues. The differentiation of neural cells is composed of steps such as cellular proliferation, neurite extension, neurite branching, synaptogenesis, and refinement of connections [3]. Modelling neuronal differentiation in a dish can provide new insights into how these connections are formed and altered. Many in vitro neuronal models are in use for genetic and pharmacologic screens such as neuronal differentiation cultures of mouse and human embryonic stem cells, induced pluripotent cells, neuronal stem cells, immortalized tumour cells (human neuroblastoma cells SH-SY5Y), NT2 human embryonal carcinoma cells, PC12 rat pheochromocytoma cells, and chicken and rodent primary neuronal cultures [4–15]. When microscopy is applied in these studies, the focus has been on changes to different morphologic parameters of neuronal cells, often in a high-throughput manner [16–24]. The use of label-free time-course phase contrast microscopy has increased our understanding on the rise, development, and maturation of neuronal networks without being confounded by factors such as phototoxicity [23, 25, 26]. Moreover, live-cell imaging with high spatial and temporal resolution has resulted in a massive increase in data volume and complexity [27, 28]. Image processing of large datasets from high throughput imaging platforms generally involve many steps of image pre-processing, segmentation, phenotype quantification and subsequent analysis [29, 30]. This stresses the need for software applications for large image data sets and automated approaches for reproducibility.

We have developed ANDA, an open-source tool for automated high-throughput image analysis of in-vitro neuronal cell cultures. ANDA is a desktop application built with TAURI that uses Python 3 scripts for data handling and function-call execution, summoning ImageJ functions from Fiji [31–33]. ANDA's main advantage is that it offers a graphical user interface that makes it easier to analyze phase contrast images with ImageJ, a process which would otherwise require extensive serial batch macro scripting that would have to be modified on a case-by-case basis.

We show that ANDA can quantify various metrics of three neuronal cell models with distinct differences in morphologies: chicken cerebellar granule neurons (CGNs), mouse primary neurons, neuronally differentiating rat PC12 cells (PC12Ns), human neuroblastoma SH-SY5Y cells (SH-SY5Ys) and pre-terminally neuronal differentiated human-derived embryonal carcinoma NTERA2 cells (NT2Ns). Decisive metrics in neuronal morphology, particularly cell bodies, neurites and neurite attachment points are retrieved and reproducibly quantified, either at single time points or in time series. ANDA is open source under the MIT license and is available on GitHub (https://github.com/EskelandLab/ANDA).

## Implementation

### Image analysis of neuronal cell types

**Fig. 1:**
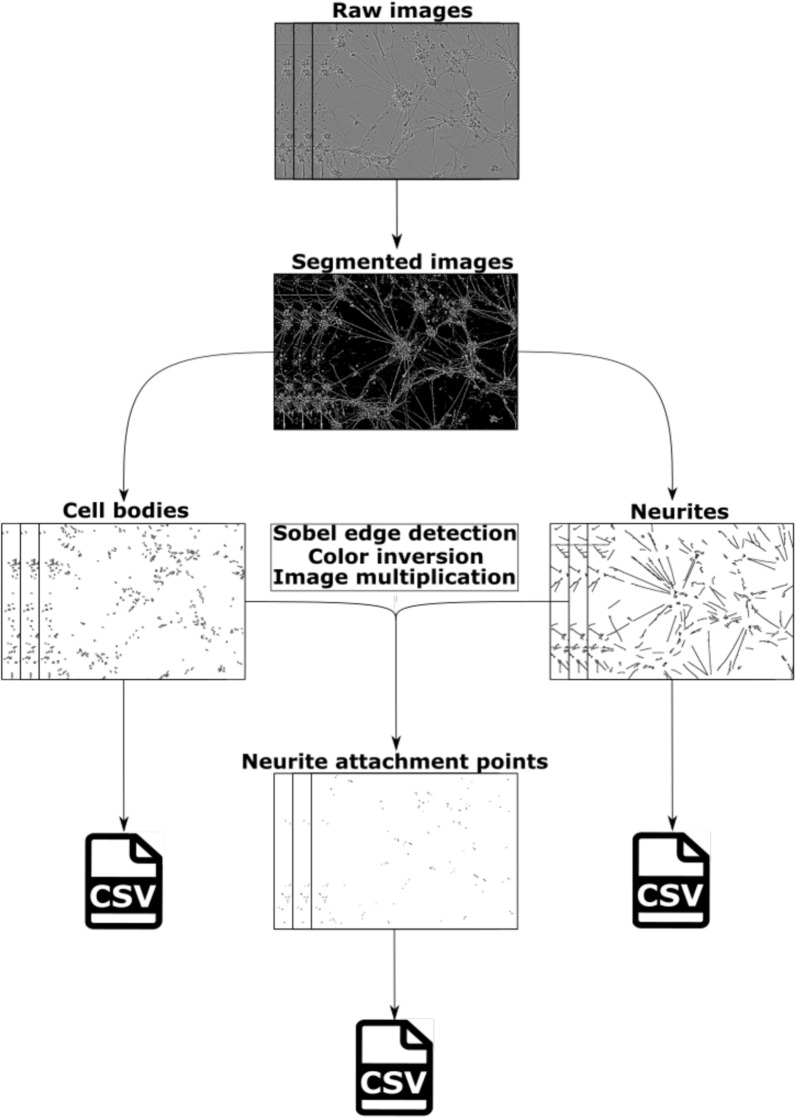
A flowchart of ANDA. Raw phase contrast images are segmented prior to identification and analysis of cell bodies or neurites. The mapping of cell bodies and neurites yield results of their own, or their outlines can be used to identify neurite attachment points. This is done by applying Sobel edge detection on the neurite outlines, followed by colour inversion of cell body outlines and neurite edges. Thereafter, the overlap between cell bodies and neurites are determined by image multiplication. These overlaps are quantified as neurite attachment points. The data is summarized in different csv files

ANDA is a tool that can measure quantity, size and shape of cell bodies, neurites, and neurite attachment points from segmented images. Furthermore, ANDA fully automates image analysis and output data summarization (Fig. 1). Prior to quantification, ANDA can be used to threshold raw images and apply different algorithms from Fiji [31] for segmentation (Additional file 1: Table S1). The pipeline also includes the option of using pre-segmented images as input. After thresholding and watershed algorithms [34] are implemented, ANDA identifies cell bodies and neurites based on segmented images. This measurement requires customization of the size and circularity of the cell bodies and neurites for the neuronal cell type of interest (Additional file 1: Table S2). Sobel edge detection is applied to identify the neurite attachment points and all output data is summarised in csv-files.

### Workflow

ANDA's workflow is designed to be straightforward and easy to customize for the user. The downstream image analysis parameters are set using a graphical user interface. Before automated image analysis, settings for cell line and neurite aspect ratio inclusion threshold have to be defined as input parameters. The images of CGNs, mouse primary neurons, NT2Ns, SH-SY5Ys and PC12Ns are successively processed and analysed in Fiji [31] and the raw output saved in csv-files. After completion of the image analysis, mean values of each selected analysis metric for each image are summarized into separate csv-files.

### Quantification of neuronal morphological metrics

ANDA presents the option to threshold images from multiple global thresholding algorithms available in Fiji [31] (Additional file 1: Table S1). In addition, the user can choose to apply a watershed algorithm to segment the images even further or use pre-segmented images as input and thereby skip a redundant segmentation step altogether. Some cell types such as the human pre-terminally differentiated NT2Ns exhibit contrast-levels that are too low to be reliably distinguished from background using standard thresholding methods, necessitating a separate segmentation-step of Weka segmentation. Weka segmentation is an unsupervised trainable machine learning algorithm that is included in Fiji, and which can improve the delineation of low-contrast objects from background, given proper training (Additional file 1: Figure S1) [35]. Following segmentation, the quantity, size and shape of cell bodies, neurites and neurite attachment points, are measured using built-in features in Fiji [31]. Cell bodies are isolated from background by applying image thresholding followed by a watershed algorithm. Similarly, neurites can be retrieved by isolating the motifs from background with thresholding and watershed algorithm. Cell bodies and neurites are quantified by identifying motifs with custom-set size and circularity criteria specified for each cell type (Additional file 1: Table S2). Neurite attachment points, a collective term for neurite trunks and neurite terminal ends, are retrieved by highlighting the edges of the neurite outlines with Sobel edge detector after thresholding and watershed algorithm, and thereafter isolating the overlap between cell body outlines and neurite outlines by colour inversion and image multiplication.

### Availability and set-up

ANDA uses brightfield images from in vitro neuronal cultures stored on disk and generates csv- files from the analysis. To be able to run ANDA download or git clone https://github.com/ EskelandLab/ANDA. For set up, example images generated and analysed in the current study can be downloaded from NeuroImaging Tools & Resources Collaboratory (NITRC) (https://www.nitrc.org/projects/anda_neuronal). Moreover, example output data for analysis has been made available for user-friendly verification of ANDA set-up.

## Results

### Qualitative measurement assessment

**Fig. 2:**
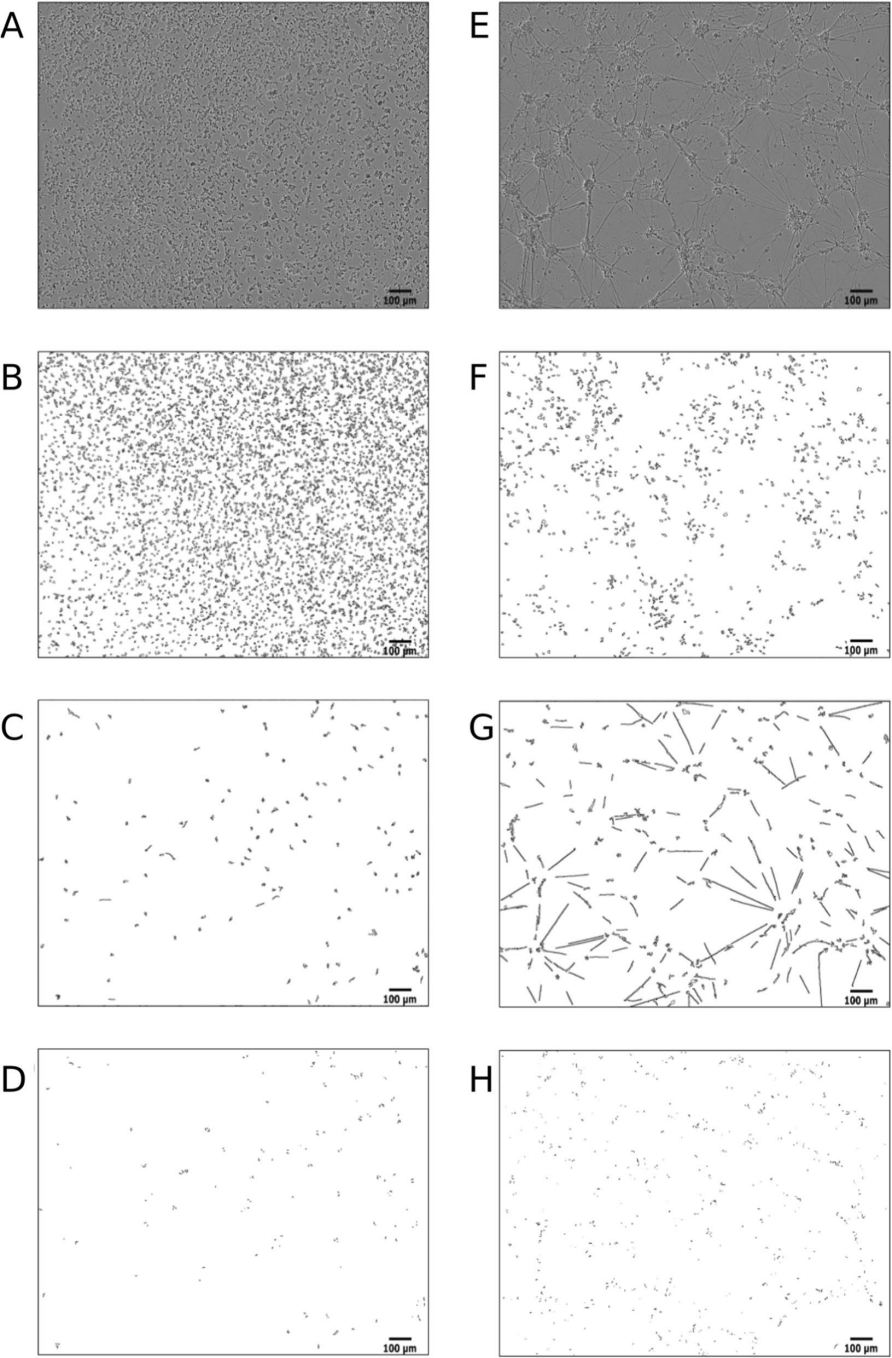
Identified cell structures from ANDA image analysis of CGN cells. **A** Phase contrast image of freshly plated cells. **B** Outlines of identified cell bodies in freshly plated cells. **C** Outlines of identified neurites in freshly plated cells. **D** Outlines of identified neurite attachment points in freshly plated cells. **E** Phase contrast of CGN cells at day in vitro (DIV) 3. **F** Outlines of identified cell bodies at DIV 3. **G** Outlines of identified neurites at DIV 3. **H** Outlines of identified neurite attachment points at DIV 3. Scale bar is 100 µm

Automated quantification of neuronal metrics from high-throughput experiments is the main purpose of our pipeline (Fig. 1). The quality of ANDA’s ability to quantify neuronal differentiation metrics was assessed using outlines of identified structures in freshly plated and in vitro day 3 CGN cells (Fig. 2), as well as freshly plated and differentiated PC12Ns (Additional file 1: Figure S2), differentiated NT2Ns (Additional file 1: Figure S3), neuroblastoma SH-SY5Y (Additional file 1: Figure S4) and in vitro day 8 E16 primary mouse neurons (Additional file 1: Figure S5). Details of the cultivation of these five cell types are described in Additional file information [18, 36, 37]. To obtain phase contrast images of the cells we used the live-cell imaging platforms IncuCyte^®^ ZOOM for the CGN and PC12N cells, and IncuCyte® S3 for the NT2N, SH-SY5Y and primary mouse neuronal cells (Additional file methods). Generally, freshly plated cells are spherical and do not exhibit neurite outgrowth until proper attachment to the growth vessel. ANDA consistently identified cell bodies in freshly plated chicken cells and in vitro day 3 (Fig. 2B, F) and mouse primary neurons in vitro day 8 (Additional file 1: Figure S5B). This trend was also observed in the PC12N, NT2N and SH-SY5Y models (Additional file 1: Figures S2B, 2E, 3B, 3E and 4B), although the NT2N cell bodies tend to have a more oblong shape.

ANDA identified CGN neurite structures at in vitro day 3 (Fig. 2G), with some artefactual identification of neurites in freshly plated cells (Fig. 2C). Similarly, neurite structures were identified in vitro day 8 mouse primary neurons (Additional file 1: Figure S5C). ANDA also detected neurite structures in three days differentiated PC12N cells but not in freshly plated cells (Additional file 1: Figures S2C, F). Some neurite structures were detected in the freshly plated NT2N cells with a clear increase after three days of differentiation (Additional file 1: Figures S3C, F). For SH-SY5Y cells cultivated for two days, some neurite structures were detected as expected for a neuronal type morphology [37]. The identification of neurite attachment points relies on identified neurite and cell body structures in the image. In freshly plated CGN and NT2N cells, ANDA falsely detected some neurites that also resulted in detection of false positive neurite attachment points (Figs. 2D and Additional file 1: Fig. S3C). True positive identification of neurite attachment points was more consistent at in vitro day 3 (Fig. 2H), NT2N day 7 neuronal differentiation (Additional file 1: Figure S3F) and mouse primary neurons (Additional file 1: Figure S5C). Based on these observations, we show that ANDA’s ability to quantify neuronal differentiation metrics improves with neuronal morphological development.

### Measurement of neuronal morphology in cell models across species

**Fig. 3:**
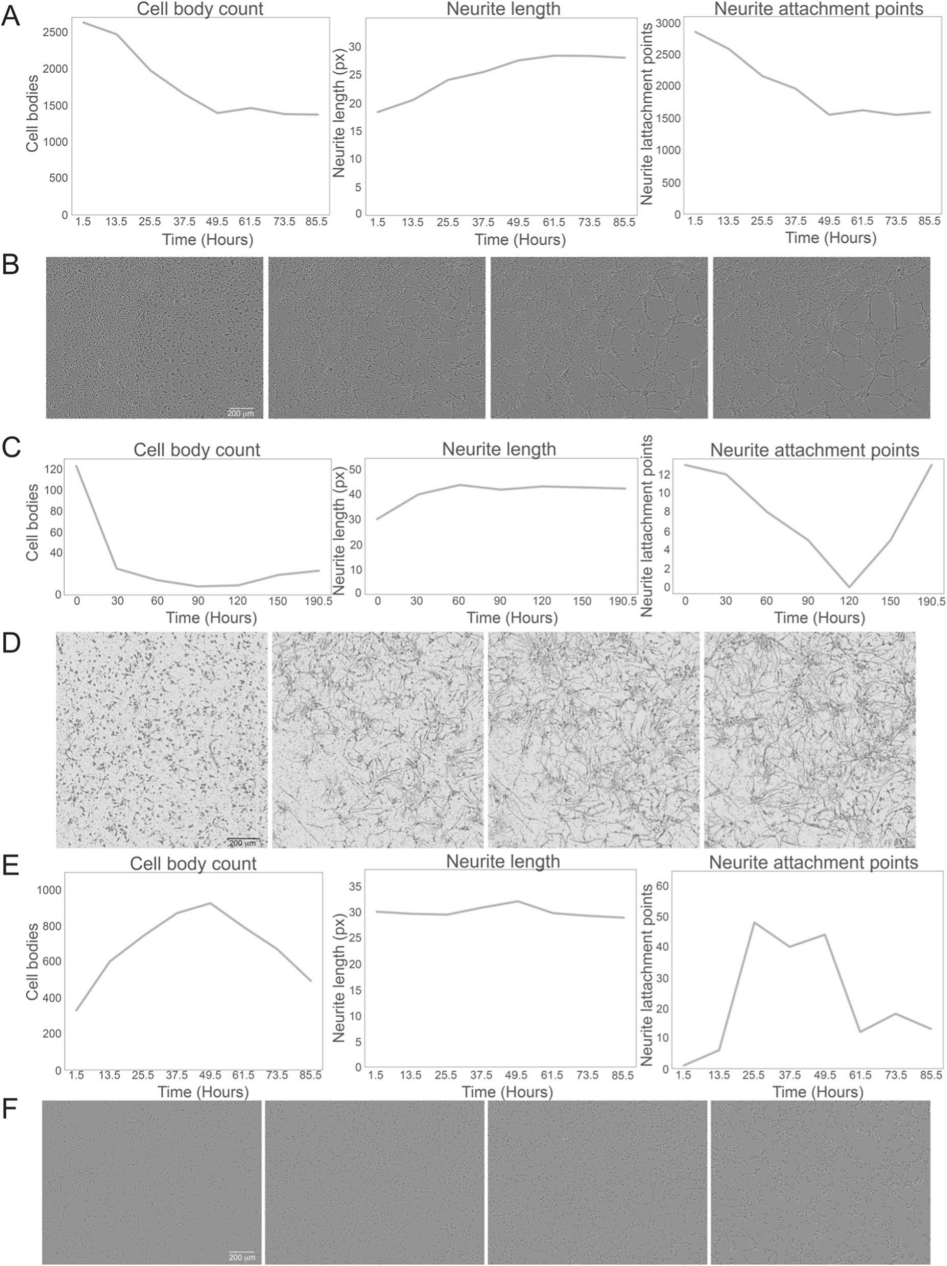
Analysis of neuronal differentiation metrics in CGNs, NT2Ns and PC12Ns. Time course measurements of neuronal differentiation in **A** CGNs, **C** NT2Ns and **E** PC12Ns. Sample phase contrast images of **B** differentiating CGNs (at 1.5, 25.5, 49.5 and 85.5 h) **D** Weka segmented images of differentiating NT2N cells (at 0, 60, 120 and 190.5 h) and **F** differentiating PC12Ns (at 1.5, 25.5, 49.5 and 73.5 h). Scale bar is 200 µm, px represents pixels

We next used ANDA to measure the morphological dynamics in chicken CGN, rat PC12N and human NT2N cells. All three models exhibit morphologies applicable for quantification of neuronal metrics. CGNs and NT2Ns exhibited a decrease in cell body count throughout differentiation, whereas PC12Ns displayed an initial slight increase up to 50 h followed by a decrease (Fig. 3). The drop in PC12Ns after 50 h can be explained by the developed dependency to nerve growth factor (NGF), and the subsequent depletion thereof in the culture media. Mean neurite lengths remained stable with a slight increase for all three models (Fig. 3A, C, E). Overall, the number of cell bodies decreased in the CGN model, however there may also be some cell bodies that cluster together that is difficult to distinguish with an automated workflow. The NT2N model exhibited decrease in numbers of cell bodies whereas cell bodies for PC12Ns showed an increase and later decreased towards the end of the experiment.

We observe that CGN cells developed longer and fewer neurites over time whereas the NT2N cells extended more and longer neurites (Fig. 3A–D). For the PC12N cells we did not detect a substantial difference, which may be because naïve PC12 cells have neurite-like filopodial protuberances that can be difficult to differentiate from neurites using our automated workflow (Fig. 3E–F). As fewer cells and neurites indicate fewer cell-to-neurite connections, the frequency of neurite attachment points also decreased in the CGNs. The number of neurite attachment points was slowly decreasing and stabilizing after approximately 50 h in the CGN cells (Fig. 3A). Similarly, the number of neurite attachment points slowly decreased but after approximately 120 h followed by a steady increase in the differentiating NT2N cells (Fig. 3C). The number of neurite attachment points curve for PC12N cells had a rapid increase followed by stabilization and decrease (Fig. 3E). Both PC12N and NT2N cells presented a low number of neurite attachment points compared to CGNs.

### Quality assessment by manual quantification

**Fig. 4:**
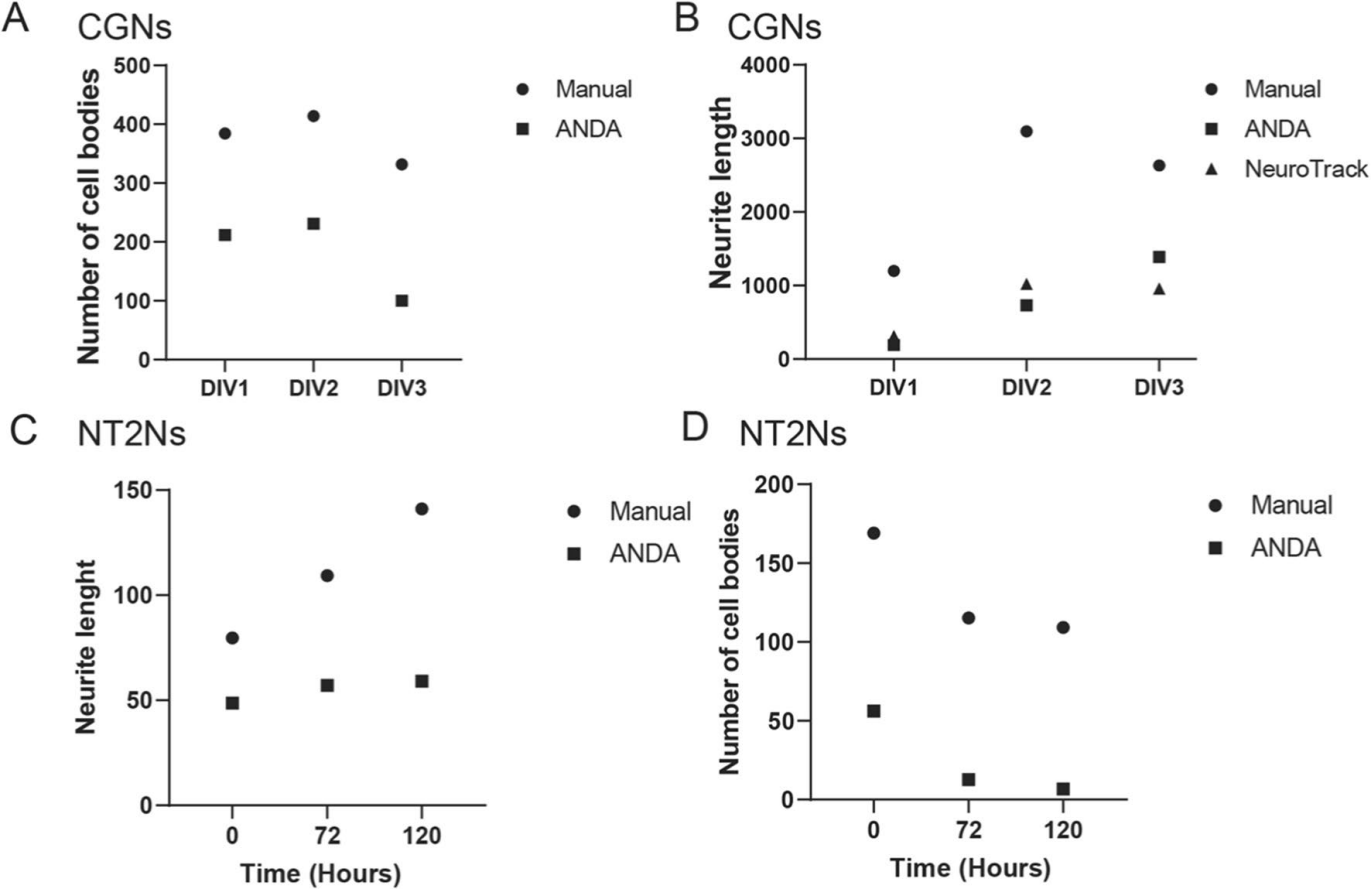
Comparison of analysis with ANDA and manual quantification of CGN and NT2N cells. **A** Comparison of output after analysis of CGN cell body counts with ANDA and manual quantification. **B** Comparison of output after analysis of CGN neurite lengths with ANDA, manual measurements and NeuroTrack. **C** Comparison of output after analysis of cell body count with ANDA and manual quantification three randomly selected areas of three differentiation time-points (0, 72 and 120 h) of NT2Ns. **D** Comparison of output after analysis of neurite lengths with ANDA and manual measurements of NT2Ns. Manual measurements for each time-point are presented as an average of two consensus counts of n = 2 randomly selected images at three stages of CGNs in vitro differentiation (DIV1, 2 and 3) and an average of two consensus counts of n = 3 randomly selected images per time-point (0, 72 and 120 h) of NT2N cell differentiation

To verify the image analysis quality of ANDA, we have used a selection of images of CGNs at early and late stages after seeding in vitro and compared the quantified output from ANDA with manual measurements (Fig. 4). The comparison between manual consensus quantification [38] and ANDA’s ability to quantify cell bodies indicates that ANDA tends to underestimate the true number of cell bodies in each image. However, both analyses showed the same trend of decreasing number of cell bodies over time (Fig. 4A). We next compared the neurite length analysis output from ANDA with manual measurements and the commercially available IncuCyte^®^ NeuroTrack Software Module (NeuroTrack) provided by Essen BioScience (Sartorius). All three analysis methods showed a similar trend in increasing mean neurite lengths over time, but both computational methods tended to underestimate the neurite lengths, especially at the later time points of CGN compared to manual counts (Fig. 4B). Next, we used a selection of images of early and late in vitro differentiated NT2Ns and compared the quantified output from ANDA with manual consensus measurements of cell bodies and neurites (Fig. 4C–D). The trends were similar to manual annotation of CGNs, ANDA underestimates the true NT2N cell numbers and neurite lengths.

**Fig. 5:**
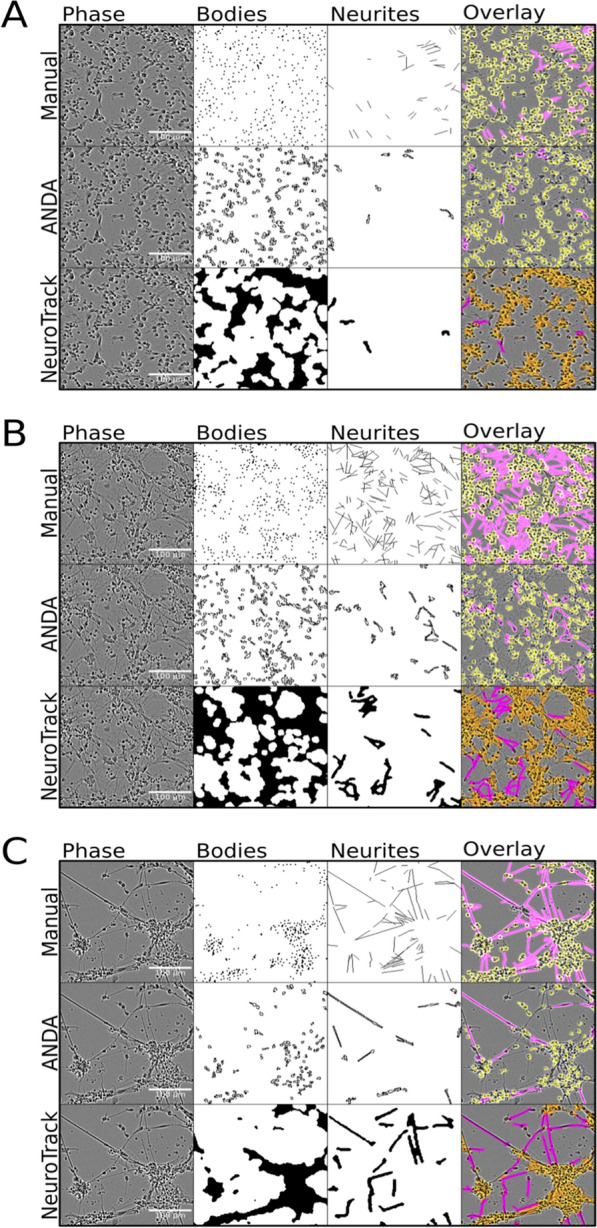
Mask comparisons between manual measurements, ANDA and NeuroTrack. From left to right: Phase contrast images, cell bodies, neurites, mask overlay. From top to bottom: manual measurements, ANDA, NeuroTrack. **A** CGNs day in vitro (DIV) 1; **B** DIV 2 and **C** DIV 3. Colours in the “overlay” panels represent cell bodies (yellow) and neurites (magenta)

Comparison of cell body analytics between ANDA and NeuroTrack could not be performed quantitatively, as NeuroTrack quantifies cells into clusters instead of attempting to delineate individual cells (Fig. 5). Judging qualitatively, both ANDA and NeuroTrack showed a tendency to detect cell bodies with minor differences to the manual measurements in the early and mid-stage time points (Fig. 5A, B). At the latest time point, NeuroTrack identified the density of cell bodies more precisely than ANDA (Fig. 5C), while yielding more false positives. The number of neurite structures was underestimated by both computational methods especially at the mid-stage time point (Fig. 5B). Although neurite counts and lengths were underestimated in comparison to the manual quantifications thereof, both ANDA and NeuroTrack tended to correctly identify oblong structures as neurites during the latest time point in CGN development (Fig. 5C).

### Time-course analysis with ANDA

**Fig. 6:**
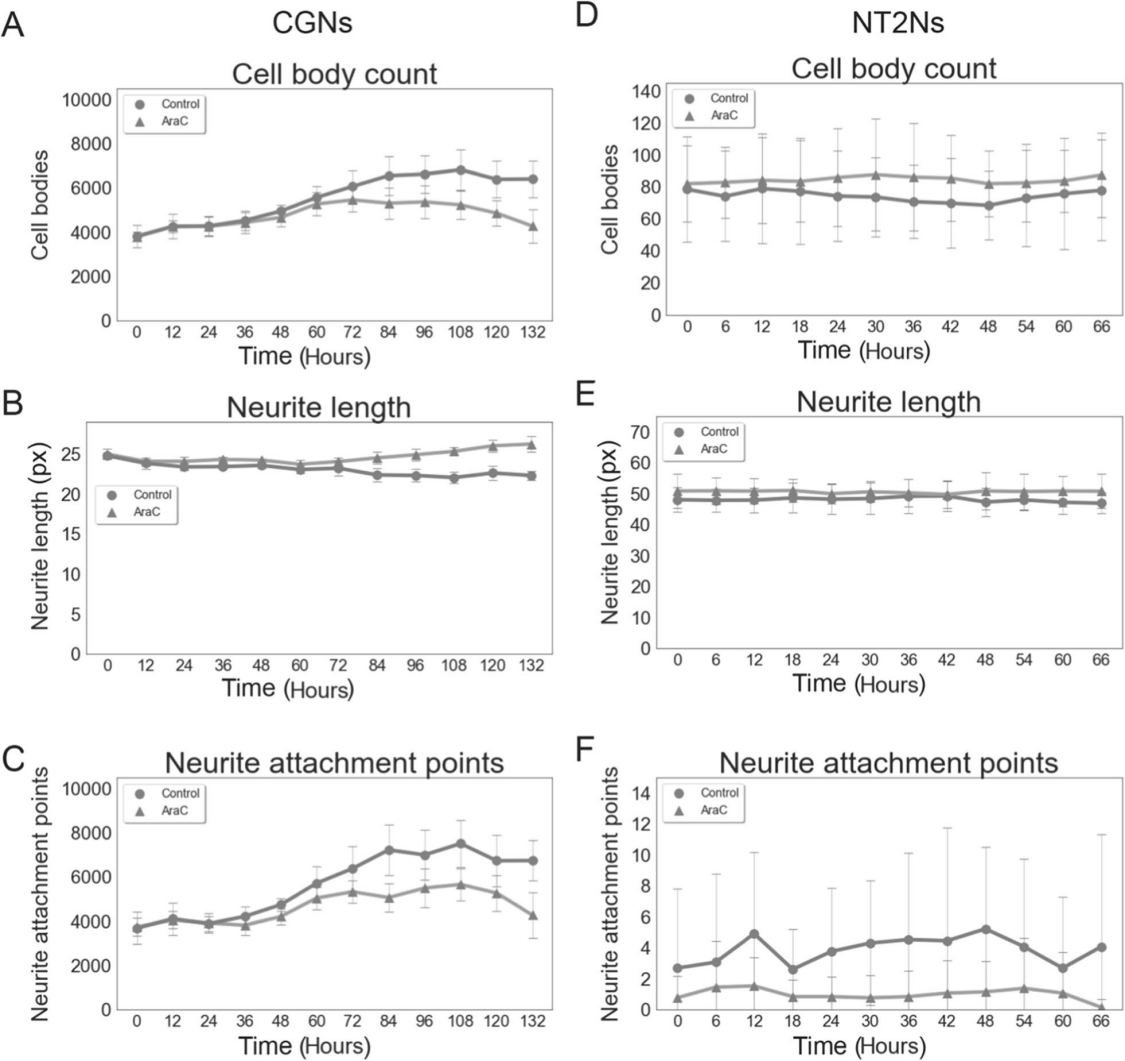
Time-course analysis of cell bodies, neurite length and attachment points in normal culture media and upon 10 µm AraC treatment by ANDA. **A** CGN cell body count, **B** CGN neurite length, **C** CGN neurite attachment points, **D** NT2N cell body count, **E** NT2N neurite length and **F** NT2N neurite attachment points. NT2N images were WEKA segmented before analysis. Each point represents average of three measurements and error bars are standard deviation. Px represents pixels

To better demonstrate the utility of ANDA for larger quantitative datasets, we set up time-course treatments with a low dose of cytosine arabinoside (AraC) in CGNs and NT2Ns (Fig. 6). AraC is a mitotic inhibitor often used as an inhibitor of glial cell proliferation in neuronal cell cultures [18]. AraC treatment is therefore expected to impact number of cell bodies over time. We performed measurements of eight areas from three individual wells during a time-course of every 12 h for a total of 132 h for CGNs (Fig. 6A–C) and every 6 h for a total of 66 h for NT2Ns (Fig. 6D–E). We observed a decrease of CGN cell number from 60 h upon AraC treatment compared to control. This was concomitant with increase in CGN neurite lengths and reduction in neurite attachment points. For NT2N no effect of AraC was observed for all three metrices, likely due to the measurement time course being too short.

We have previously shown that ANDA is able to quantify the impact CGN and NT2N neurite lengths and neurite attachment points upon treatment with increasing doses of the analgesic paracetamol (acetaminophen) [18]. After 72 h, lower doses of paracetamol had no significant effect on NT2Ns, whereas the highest dose showed an effect on neurite length and neurite attachment points.

## Discussion

ANDA is an image analysis tool particularly useful for analysis of large datasets from live cell high-throughput neuronal image time-series and drug exposures [18]. The comparison of the metrics of the five different in vitro cell models suggests that the numerical output from analysis with ANDA is relatively consistent with what is observed in the images. In comparing ANDA to NeuroTrack, the two methods yielded relatively similar outputs. However, whereas NeuroTrack averages cell body counts into clusters, ANDA quantifies individual soma, including somatic characteristics such as size and shape, yielding a much higher granularity of data. The discrepancies between the numerical output and what is observed in the images can to a large degree be attributed to identification of false positive structures such as dead cell debris or structures falsely quantified due to over segmentation of the images. As with any automated method, ANDA comes with some limitations.

These limitations are mostly dependent on the quality of the data, with higher quality reducing the possibility of errors by image pre-processing and segmentation. Therefore, we have outlined some recommendations for image analysis to improve the quality of the output. For any new cell type to be analysed, we recommend doing a manual consensus quantification to set up the ANDA analysis. The experimental setup should ensure that cell densities do not become excessively high. As ANDA is not performing uncertainty assessment, we recommend the use of images with notable contrast between cells and background. If low-contrast cells are used, as described here for NT2Ns, we strongly recommend Weka-based pre-segmentation prior to analysis with ANDA [35]. At too high cell densities ANDA is not able to correctly segment cells from each other or the background. Furthermore, ANDA is not able to distinguish individual neurites in fasciculated neurite bundles and will only retrieve the length of the identified oblong objects, regardless of its width. False positive neurites can be identified by comparing image outlines with the raw data. ANDA also includes the option to set an aspect ratio threshold for which oblong structures are regarded as false positive or not. Some primary cells, glial cells and neuronal cells cannot be clearly distinguished by phase contrast alone. The user can, however, isolate granule cells from the images based on criteria such as size and shape.

We have shown that ANDA was able to capture the overall trend from the images represented here, albeit with a subtle over- or under-estimation of the number of neuronal metrics (Fig. 3). To address this, we have included an option to remove falsely identified neurites from the final output based on neurite aspect ratio in ANDA. This can be achieved by setting a certain threshold. All neurite structures with an aspect ratio below this will be regarded as false positive and therefore not included in the final output when mean neurite length and number of neurite attachment points are summarized.

## Conclusion

We have demonstrated that the open-source tool ANDA is suitable for analysis of high-throughput images of differentiating neuronal cells from human, mouse, rat, and chicken in vitro models. ANDA can effectively analyse time-series image sets of differentiating neuronal cells with vastly differing morphologies. To that end, we have shown that ANDA is an accurate, versatile, efficient, and user-friendly tool for quantification of neuronal morphometrics in different model systems.

*Project name* ANDA: An open-source tool for automated image analysis of neuronal differentiation.

*Project home page* https://github.com/EskelandLab/ANDA and https://www.nitrc.org/projects/anda_neuronal/

*Operating system(s)* Linux, MacOS, Windows.

*Programming language* Python, shell, HTML, CSS, JavaScript, Rust.

*License* MIT.

*Any restrictions to use by non-academics* MIT.

### Supplementary Information

**Additional file 1: Figure S1.** NT2N cells before and after segmentation with Weka. **Figure S2.** Identified cell structures from ANDA image analysis of PC12N cells. **Figure S3.** Identified cell structures from ANDA image analysis of Weka segmented NT2N cells. **Figure S4. **Identified cell structures from ANDA image analysis of SH-SY5Y cells **Figure S5.** Identified cell structures from ANDA image analysis of E16 DIV 8 mouse primary neurons. **Table S1.** Fiji [1] and built-in features used for image analysis. **Table S2.** Size and shape criteria used for analysis of NT2Ns, CGNs, PC12Ns SHSY5Y cells, and mouse primary neurons for ANDA.

## Data Availability

Example datasets generated and analysed during the current study are available as downloads in the NeuroImaging Tools & Resources Collaboratory (NITRC) https://www.nitrc.org/projects/anda_neuronal.
