# Supplementary material for: ANDA: an open-source tool for automated image analysis of in vitro neuronal cells
Source: BMC Neurosci. 2023 Oct 24;24:56. doi: 10.1186/s12868-023-00826-z (PMC10594822; doi:10.1186/s12868-023-00826-z)
Supplement: Supplementary file 1 — Additional file 1: Figure S1. NT2N cells before and after segmentation with Weka. Figure S2. Identified cell structures from ANDA image analysis of PC12N cells. Figure S3. Identified cell structures from ANDA image analysis of Weka segmented NT2N cells. Figure S4. Identified cell structures from ANDA image analysis of SH-SY5Y cells Figure S5. Identified cell structures from ANDA image analysis of E16 DIV 8 mouse primary neurons. Table S1. Fiji [1] and built-in features used for image analysis. Table S2. Size and shape criteria used for analysis of NT2Ns, CGNs, PC12Ns SHSY5Y cells, and mouse primary neurons for ANDA. [file 12868_2023_826_MOESM1_ESM.pdf]

Additional file information

ANDA: An open-source tool for automated image analysis of in vitro neuronal cells

Hallvard Austin Wæhler[1,4,5], Nils-Anders Labba[1,2,4], Ragnhild Elisabeth Paulsen[2,4], Geir Kjetil Sandve[3,4] and Ragnhild Eskeland[1,4,5#]

[1]Institute of Basic Medical Sciences, Department of Molecular Medicine, Faculty of Medicine, University of Oslo, PO Box 1112 Blindern, 0317 Oslo, Norway

[2] Section for Pharmacology and Pharmaceutical Biosciences, Department of Pharmacy, University of Oslo, 0316 Oslo, Norway

[3] Department of Informatics, University of Oslo, 0316 Oslo, Norway

[4] PharmaTox Strategic Research Initiative, Faculty of Mathematics and Natural Sciences, University of Oslo, 0316 Oslo, Norway

[5] Centre for Cancer Cell Reprogramming, Institute of Clinical Medicine, Faculty of Medicine, University of Oslo, 0317 Oslo, Norway

# Correspondence to: ragnhild.eskeland@medisin.uio.no

Additional file Figures

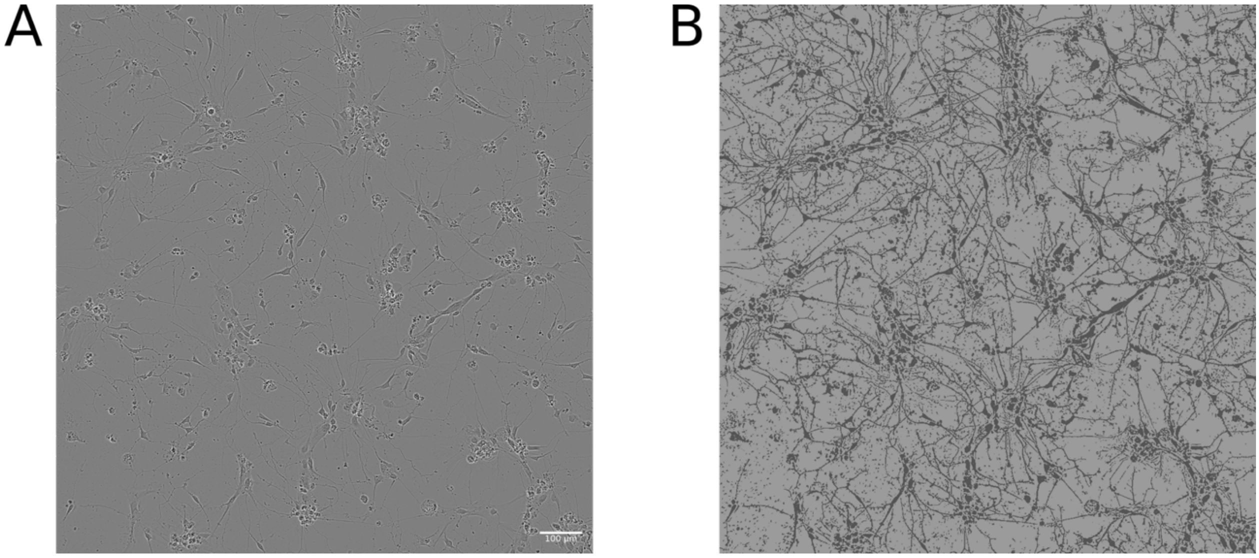

Additional file Figure 1. NT2N cells before and after segmentation with Weka. A: Phase contrast image of 190,5 hour differentiated NT2N obtained from Incucyte® S3. B: The same image after Weka segmentation. Scale bar is 100 µm.

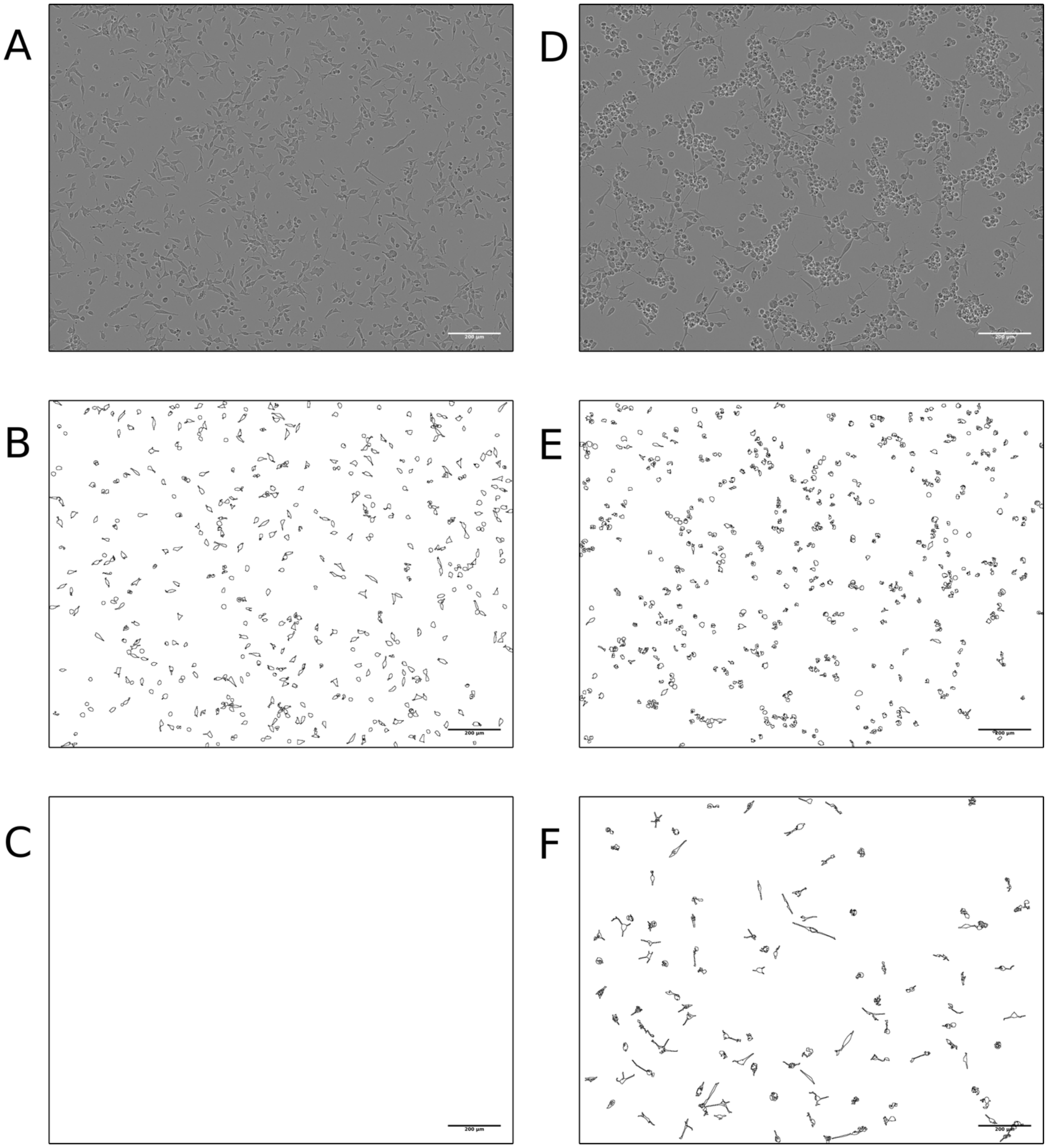

Additional file Figure 2. Identified cell structures from ANDA image analysis of PC12N cells. (A) Phase contrast image of freshly plated cells. (B) Outlines of identified cell bodies in freshly plated cells. (C) Freshly plated PC12N cells do not show developed neurites and, therefore, ANDA did not identify neurites. (D) Phase contrast of PC12N cells at day 3. (E) Outlines of identified cell bodies at day 3. (F) Outlines of identified neurites at day 3. Scale bar is 200 micrometres.

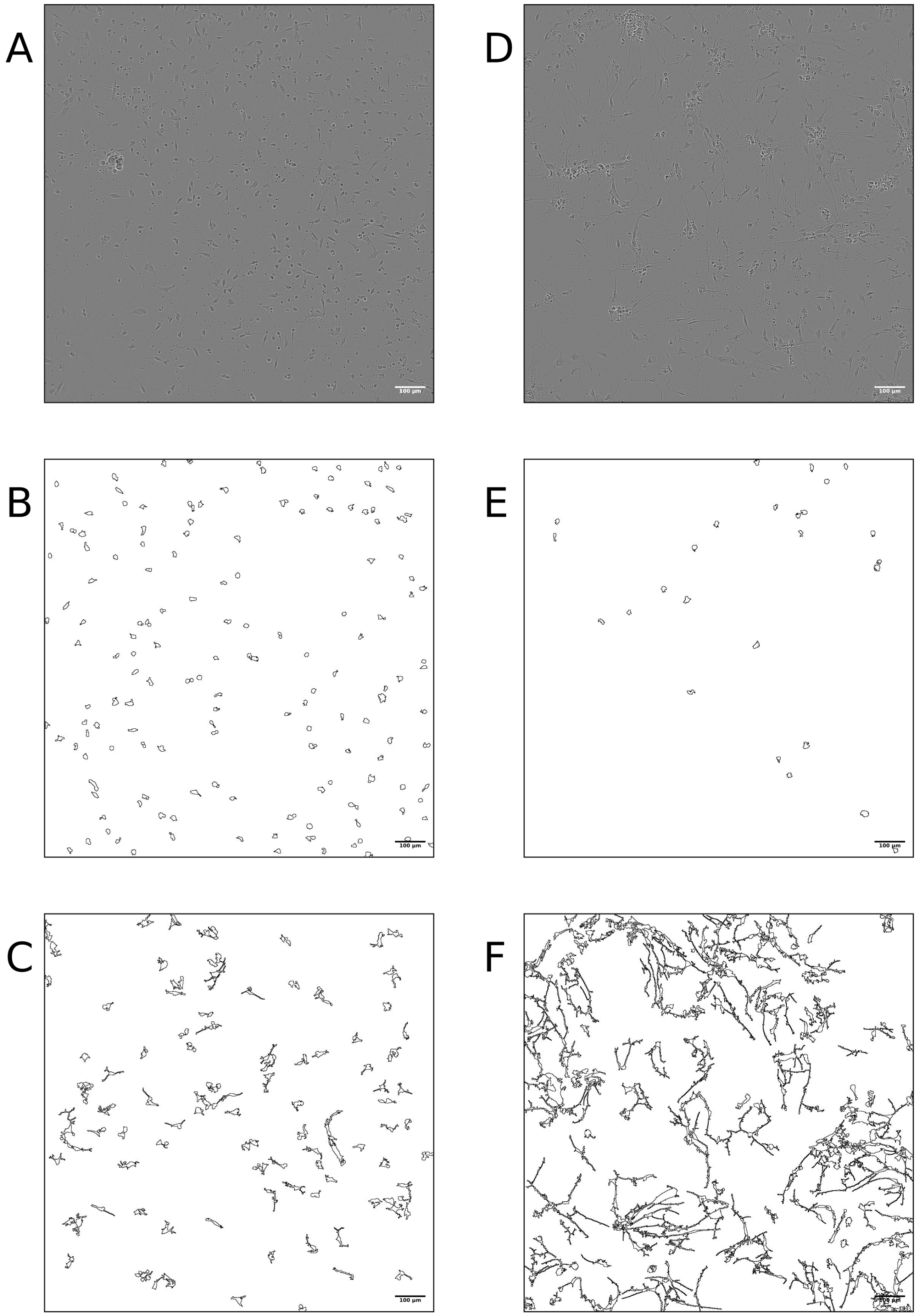

Additional file Figure 3. Identified cell structures from ANDA image analysis of Weka segmented NT2N cells. (A) Phase contrast image of freshly plated cells. (B) Outlines of identified cell bodies in freshly plated cells. (C) Outlines of identified neurites in freshly plated cells. (D) Phase contrast image at neuronal differentiation day 7. (E) Outlines of identified cell bodies at day 7. (F) Outlines of identified neurites at day 7. Scale bar is 100 micrometres.

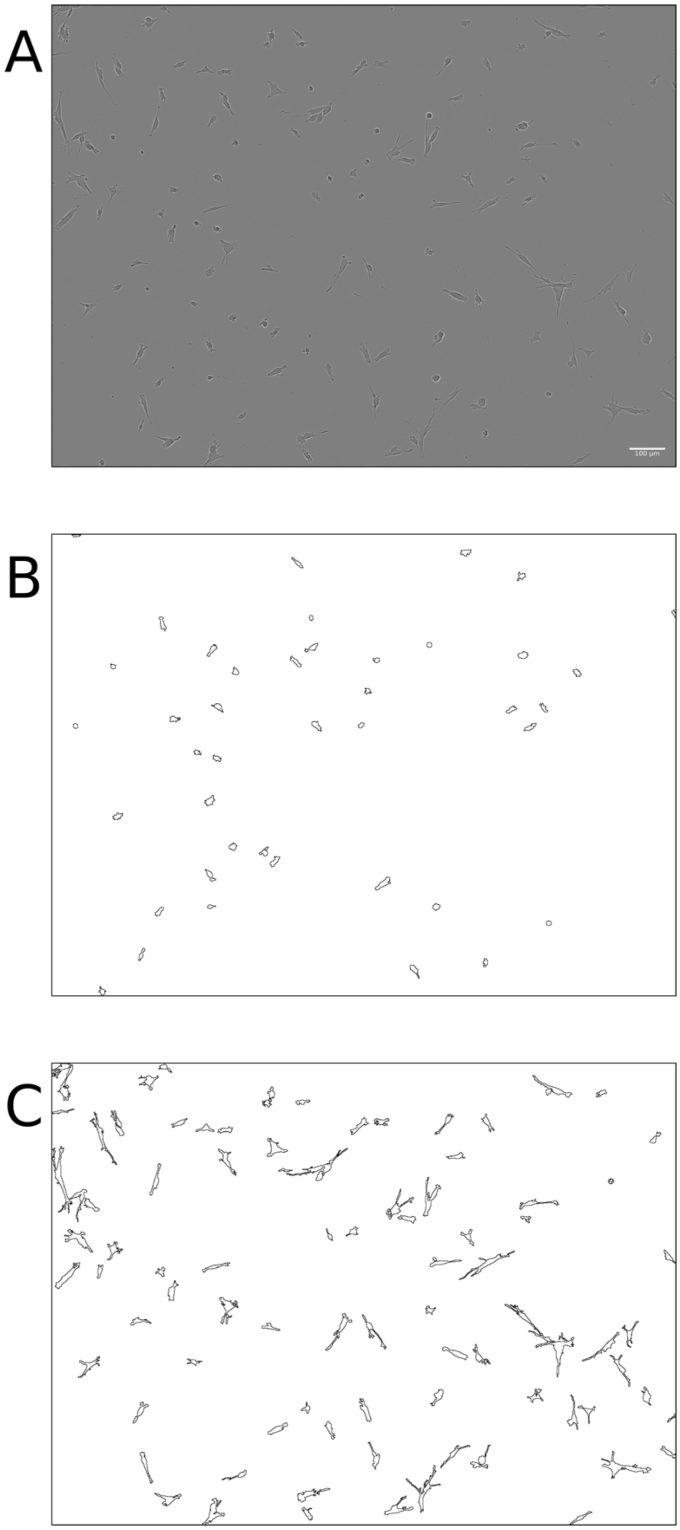

Additional file Figure 4. Identified cell structures from ANDA image analysis of SH-SY5Y cells. (A) Phase contrast image of SH-SY5Y cells. (B) Outlines of identified cell bodies. (C) Outlines of identified neurites. Scale bar is 100 micrometres.

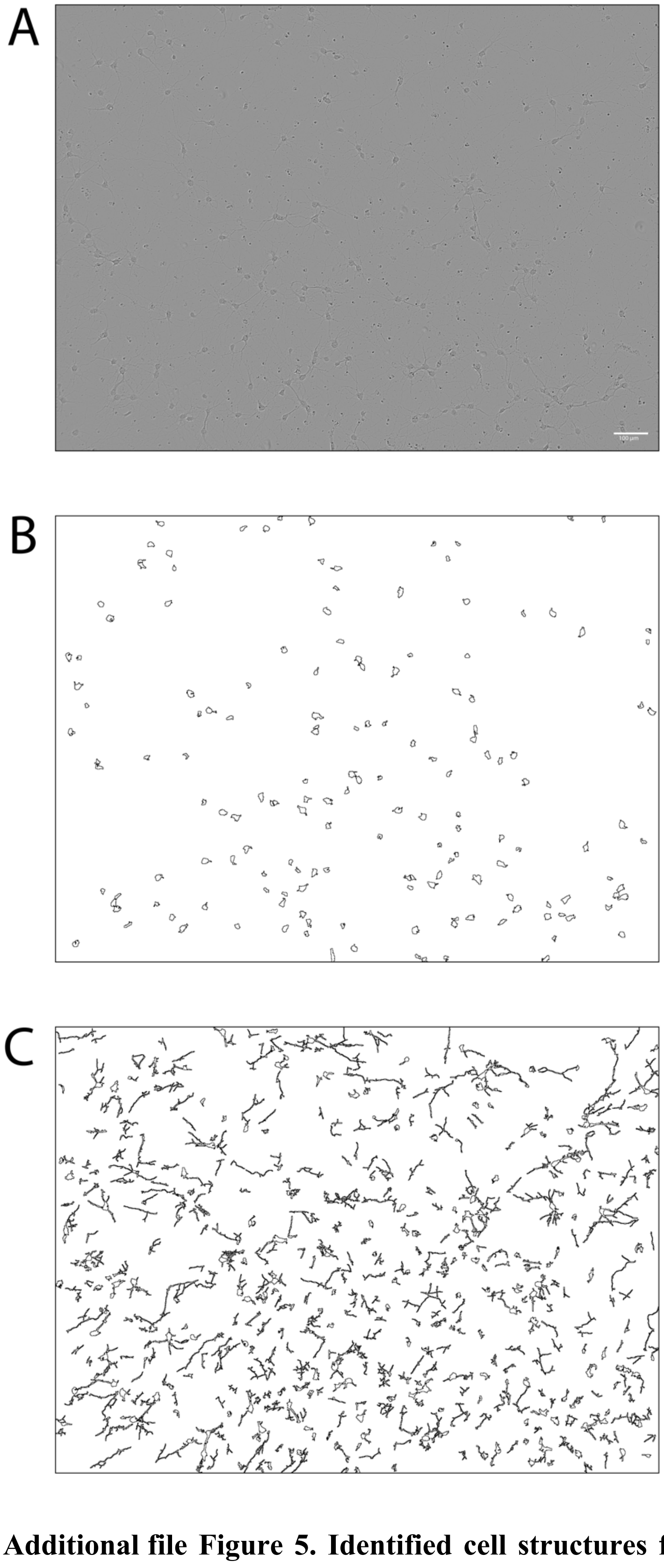

Additional file Figure 5. Identified cell structures from ANDA image analysis of E16 DIV 8 mouse primary neurons. (A) Phase contrast image of eight days in vitro cells. (B) Outlines of identified cell bodies eight days in vitro cells. (C) Outlines of identified neurites eight days in vitro cells. Scale bar is 100 micrometres.

Additional file Table

Supplementary Table 1. Fiji[1] and built-in features used for image analysis

Built-in feature	Web links
Fiji	https://imagej.net/software/fiji/
IJ.java	
ParticleAnalyzer.java	
ImageCalculator.java	
ImagePlus.java	
AutoThresholder.java	
Converter.java	
Watershed_Algorithm.jar	https://imagej.net/plugins/watershed-algorithm

Additional file Table 2. Size and shape criteria used for analysis of NT2Ns, CGNs, PC12Ns SH-SY5Y cells, and mouse primary neurons for ANDA.

	Min. circularity	Max. circularity	Min. size (px)*	Max. size (px)*
	Cell bodies			
NT2Ns	0.38	1.00	90	900
CGNs	0.40	1.00	16	175
PC12Ns	0.20	1.00	60	175
SH-SY5Y	0.32	1.00	300	1450
Mouse primary neurons	0.38	1.00	300	1450
	Neurites			
NT2Ns	0.00	0.38	200	1800
CGNs	0.00	0.38	89	1249
PC12Ns	0.00	0.22	160	1000
SH-SY5Y	0.00	0.32	70	1000
Mouse primary neurons	0.00	0.32	70	1000

*Px = pixels.

Additional file methods

Chicken cerebellar granule neuronal cells

The generation of chicken granule neuronal cells was performed as described previously [2]. Fertilized chicken eggs (*Gallus gallus*) were incubated at 37.5 °C, 45 % humidity for 17 days, anaesthetized and sacrificed prior to cerebellar excision (approved under Norwegian Food Safety Authority FOTS ID 13896). CGN culture was prepared by trypsinization and trituration of pooled cerebella, followed by seeding in Basal Eagle's Medium supplemented with chicken serum into PLL-coated 96-well plates at 530.000 cells/ cm^2. Following overnight incubation, serum-containing medium was replaced with defined medium supplemented with 10 μM β-D-arabinofuranoside (AraC, Sigma-Aldrich #C1768) to limit glial proliferation.

Primary mouse neuronal culture

Mouse primary neuronal cells were isolated from C57BL/6J embryos (E16) (approved by Norwegian Food Safety Authority (project no. 30120) in accordance with the guidelines of the Section of Comparative Medicine at the Institute of Basic Medical Sciences, University of Oslo). Embryos were washed in DPBS A (DPBS, 0.5 mM Glutamax I supplement (ThermoFischer) and 1x Penstrep (Fisher Scientific)). Cerebral cortex was dissected, and meninges and blood vessels removed. The tissue was cut into pieces in complete medium (NeuroBasal Plus medium (ThermoFischer), 1x B27 supplement (ThermoFischer), 0.5 mM Glutamax I and 1x Penstrep) and prepared by trypsinization followed by a 10 minute treatment with 0.25mg/ml DNaseI (Sigma) at room temperature. The cells were washed twice in complete medium and filtered through a 70 mm nylon cell strainer. Cells were plated in PDL-coated plates at 50 000 cells/cm^2 and incubated at 37.5 °C, in 5 CO_2 % and 45 % humidity for 8 days with half media change every 3-4 days.

In vitro differentiation of NTERA2 and PC12

The generation of neuronal NT2N cells (ATCC #CRL-1973) was performed as described previously [2]. Briefly, human NTERA2 embryonal carcinoma cells (ATCC) were seeded into 100 mm bacterial dishes at 5×10^5 cells/mL in 10 mL Dulbecco's modified Eagle's medium supplemented with 10% foetal bovine serum and incubated at 37°C with 5 % CO_2 on a rotator for 2-4 days until spheroids formed. Spheroids were differentiated in rotary culture in serum-containing medium supplemented with 10 µM retinoic acid (ThermoFischer #R2625) for 6 days, with half-volume medium change taking place every two days. On day 6, serum-containing medium was replaced with defined medium consisting of DMEM/F12 supplemented with B27 and N2 (according to manufacturer's instructions), as well as 10 µM retinoic acid, in which the spheroids were differentiated in rotary culture for another 14 days, with half-volume medium changes taking place every two days. Spheroids were then trypsinized and seeded into PLL- and geltrex-coated 96-well plates at 50 000 cells/cm^2 in 1:1 mixture of conditioned and fresh defined medium supplemented with 10 µM retinoic acid.

The rat pheochromocytoma cell line PC12 (ATCC #CRL-1721) was cultivated in DMEM supplemented with 5% horse serum and 10% foetal bovine serum as described previously [3]. Cells were seeded into 96-well plates at a density of 8 x 10^3 cells/cm^2. Following overnight incubation, medium was replaced with fresh DMEM containing 2 % horse serum and 50 ng/mL nerve growth factor for three days.

Cultivation of SH-SY5Y

The human neuroblastoma SH-SY5Y cells (ATCC #CRL-2266)[4] were seeded into 96 well plates at 3000 cells per well in high-glucose DMEM supplemented with GlutaMAX and pyruvate (ThermoFisher) and allowed to proliferate for 2 days in a humidified incubator with 5 % CO_2 at 37 °C prior to being imaged.

AraC treatment of CGNs and NT2Ns

Treatment of CGNs and NT2Ns was conducted by addition of defined medium described above supplemented with 10 μM β-D-arabinofuranoside (AraC, Sigma-Aldrich #C1768) prior to imaging in intervals of 6 or 12 hours. In cases where cells were imaged past 72 hours, media were changed every 3 days, with treatments being refreshed at half of initial concentration, where applicable.

Imaging with Incucyte®

Live-cell imaging was performed at 37 °C and 5% CO_2 using the Incucyte® ZOOM and Incucyte® S3 platforms (EssenBioScience, Hertfordshire, UK). CGN and PC12N experiments were carried out in TPP® 96-well plates (Sigma-Aldrich #Z707910) in Incucyte® ZOOM, whereas NT2N experiments were undertaken in Corning® black-frame clear-bottom 96-well plates (Corning #3603) in Incucyte® S3. Phase contrast images were acquired at 10x magnification using the Incucyte®'s built-in settings corresponding to each plate type.

Manual count and quantification with Neurotrack

To evaluate the performance of ANDA, its outputs were compared to the outputs of the EssenBioscienceIncucyte® ZOOM Neurotrack Analysis Software Modul, as well as to manual quantifications. Two randomly selected areas of three sets of images of CGNs from DIV1, DIV2, and DIV3 were analysed using the three different modalities. Manual quantifications were performed by two expert personnel, where cell soma and neurites were manually annotated, followed by discussion of the results of the two annotation-sets in order to reach a consensus-annotation[5]. The consensus-annotation was then quantified using the particle analysis and skeleton analysis modules of FIJI/ImageJ. The Neurotrack quantification module was trained using a set of analysis parameters optimized based on a training set from the CGN cells consisting of three images per time-point at 6, 24, 48, and 72 h post-seeding. Analysis with ANDA was executed using the CGN analysis mode. For

manual counts of NT2Ns, three randomly selected areas of three sets of NT2Ns from differentiation day 1, 3, and 6 were annotated and quantified as described above.

Requirements for ANDA

To be able to use ANDA, the user needs to have and Python 3 installed [6].

References

1. Schindelin J, Arganda-Carreras I, Frise E, Kaynig V, Longair M, Pietzsch T, et al. Fiji: an open-source platform for biological-image analysis. Nat Methods. 2012;9:676–82.

2. Labba N-A, Wæhler HA, Houdaifi N, Zosen D, Haugen F, Paulsen RE, et al. Paracetamol perturbs neuronal arborization and disrupts the cytoskeletal proteins SPTBN1 and TUBB3 in both human and chicken in vitro models. Toxicology and Applied Pharmacology. 2022;449:116130.

3. Rakkestad KE, Sørvik IB, Øverby GR, Debernard KAB, Mathisen GH, Paulsen RE. 17α-Estradiol down-regulates glutathione synthesis in serum deprived PC-12 cells. Free Radical Research. 2014;48:1170–8.

4. Kovalevich J, Langford D. Considerations for the Use of SH-SY5Y Neuroblastoma Cells in Neurobiology. Methods Mol Biol. 2013;1078:9–21.

5. Giovannucci A, Friedrich J, Gunn P, Kalfon J, Brown BL, Koay SA, et al. CaImAn an open source tool for scalable calcium imaging data analysis. eLife. 2019;8:e38173.

6. Van Rossum G, Drake FL. Python 3 Reference Manual. Scotts Valley, CA: CreateSpace.; 2009.
